# Development and Interpretation of a Clinicopathological-Based Model for the Identification of Microsatellite Instability in Colorectal Cancer

**DOI:** 10.1155/2023/5178750

**Published:** 2023-02-18

**Authors:** Zhenxing Jiang, Lizhao Yan, Shenghe Deng, Junnan Gu, Le Qin, Fuwei Mao, Yifan Xue, Wentai Cai, Xiu Nie, Hongli Liu, Fumei Shang, Kaixiong Tao, Jiliang Wang, Ke Wu, Yinghao Cao, Kailin Cai

**Affiliations:** ^1^Department of Gastrointestinal Surgery, Union Hospital, Tongji Medical College, Huazhong University of Science and Technology, Wuhan, Hubei 430022, China; ^2^Department of Hand Surgery, Union Hospital, Tongji Medical College, Huazhong University of Science and Technology, Wuhan 430022, China; ^3^Department of General Surgery, First Affiliated Hospital, School of Medicine, Shihezi University, Shihezi, Xinjiang 832008, China; ^4^College of Life Science and Technology, Huazhong University of Science and Technology, Wuhan, Hubei 430022, China; ^5^Department of Pathology, Union Hospital, Tongji Medical, Huazhong University of Science and Technology, Wuhan, Hubei 430022, China; ^6^Cancer Center, Union Hospital, Tongji Medical College, Huazhong University of Science and Technology, Wuhan 430022, China; ^7^Department of Medical Oncology, Nanyang Central Hospital, Nanyang, Henan, China; ^8^Department of Digestive Surgical Oncology, Cancer Center, Union Hospital, Tongji Medical College, Huazhong University of Science and Technology, Wuhan 430022, China

## Abstract

Chemotherapy is not recommended for patients with deficient mismatch repair (dMMR) in colorectal cancer (CRC); therefore, assessing the status of MMR is crucial for the selection of subsequent treatment. This study is aimed at building predictive models to accurately and rapidly identify dMMR. A retrospective analysis was performed at Wuhan Union Hospital between May 2017 and December 2019 based on the clinicopathological data of patients with CRC. The variables were subjected to collinearity, least absolute shrinkage and selection operator (LASSO) regression, and random forest (RF) feature screening analyses. Four sets of machine learning models (extreme gradient boosting (XGBoost), support vector machine (SVM), naive Bayes (NB), and RF) and a conventional logistic regression (LR) model were built for model training and testing. Receiver operating characteristic (ROC) curves were plotted to evaluate the predictive performance of the developed models. In total, 2279 patients were included in the study and were randomly divided into either the training or test group. Twelve clinicopathological features were incorporated into the development of the predictive models. The area under curve (AUC) values of the five predictive models were 0.8055 for XGBoost, 0.8174 for SVM, 0.7424 for NB, 8584 for RF, and 0.7835 for LR (Delong test, *P* value < 0.05). The results showed that the RF model exhibited the best recognition ability and outperformed the conventional LR method in identifying dMMR and proficient MMR (pMMR). Our predictive models based on routine clinicopathological data can significantly improve the diagnostic performance of dMMR and pMMR. The four machine learning models outperformed the conventional LR model.

## 1. Introduction

Colorectal cancer (CRC) is one of the most common cancers worldwide and the second leading cause of cancer-related deaths [1]. Deficient mismatch repair (dMMR) is presented in 10–20% of CRC cases, suggesting that CRC with dMMR is a biologically distinct type of CRC with broad prognostic, predictive, and therapeutic importance [2]. Furthermore, the DNA MMR system is an evolved and conserved process for repairing errors during replication in proliferating cells [3]. Molecularly targeted therapies and chemotherapeutic agents are used to treat patients with dMMR CRC [4]. Recently, a growing body of evidence suggests that the individual treatment response of patients with CRC is strongly related to its molecular characteristics [5].

Microsatellite instability (MSI) is the abnormal shortening or lengthening of 1–6 repeat base pair units of DNA caused by inactivation of the MMR system [3, 6, 7]. The patients with CRC presenting with MSI are more likely to have Lynch syndrome [8, 9]. Thus, MMR is essential to ensure the stability of genetic information and avoid future genetic diseases [10]. According to the National Comprehensive Cancer Network (NCCN), patients with stage II CRC with MSI or dMMR did not require chemotherapy, which is beneficial for many patients with CRC. A study by Klingbiel et al. [11] and Overman et al. [12] showed that patients with CRC and MSI are insensitive to pentafluorouracil chemotherapy but sensitive to PD-1 immunotherapy, which provides more rationalization of CRC treatment. However, most patients are unable to undergo genetic testing to detect the dMMR status due to cost and time constraints.

Recently, artificial intelligence has become a research hotspot in medicine due to the potential to achieve high-precision automated diagnosis of heterogeneous diseases. Skrede et al. [13] used deep learning combined with conventional digital scanning of hematoxylin and eosin- (HE-) stained tumor tissue sections to develop a clinically useful prognostic marker that can classify stage II and III patients into different prognostic groups and then guide the application of adjuvant chemotherapy. Howard et al. [14] used a machine learning model to successfully predict which of the patients, among those who underwent surgery, would require the removal of squamous cell carcinoma of the neck and who, being at intermediate risk, would benefit from receiving cisplatin-based chemoradiation therapy (CRT). Lai et al. [15] found that artificial intelligence predicted the survival rate following liver cancer treatment with higher accuracy than did the traditional linear analysis systems. In addition, Yu et al. [16] applied seven machine learning classifiers to predict the survival time of patients with lung cancer based on histopathological features with satisfactory prediction accuracy. However, no study has systematically evaluated the detection value of machine learning models based on simple clinicopathological indicators for dMMR. Therefore, a simple minimally invasive accurate method for identifying dMMR is urgently required.

Based on simple clinicopathological indicators and with reference to previous studies, four machine learning models and a logistic regression model were developed in this study to predict CRC lacking DNA MMR, aid clinicians in identifying MMR status, and provide a reference for a precise treatment plan for patients.

## 2. Materials and Methods

### 2.1. Study Population

Retrospective analysis of 2279 patients of CRC with confirmed diagnosis at Wuhan Union Hospital from May 2017 to December 2019 was done. Patients with the following conditions were excluded from the study: (i) no MMR status outcome, (ii) no complete clinical data, and (iii) history of radiotherapy and chemotherapy prior to MMR status identification. A total of 2279 patients were enrolled in our study and randomly assigned to train and test sets in a 7-to-3 ratio. The consensus criteria for dMMR protein diagnosis were to select CRC patients who met the Revised Bethesda Guidelines (RBG) and then underwent MSI testing and/or immunohistochemical staining for MMR protein. This study was approved by the Ethics Committee of Union Hospital, Tongji Medical College, Huazhong University of Science and Technology (No. 2018-S377). All patients signed an informed consent form stating that they understood the procedure and its potential complications and agreed to participate in this study.

### 2.2. Data Collection

Baseline clinicopathological information on the patients obtained from the hospital's medical records included the following serum tumor markers: carcinoembryonic antigen (CEA), glycoantigen 19-9 (CA19-9), glycoantigen 12-5 (CA12-5), glycoantigen 72-4 (CA72-4), glycoantigen 15-3 (CA15-3), alpha-fetoprotein (AFP), serum squamous cell carcinoma antigen (SCC), ferritin (FERR), cytokeratin 19 fragment cyfra21-1 (CYFRA21-1), serum neuron-specific enolase (NSE), pathological type, histological type, age, sex, location, diameters, number of sampled lymph nodes (LNs), number of positive LNs, T-stage, N-stage, M-stage, perineural invasion, and vascular invasion. MMR status was assessed by immunohistochemistry (IHC) and was determined by MSH2, MSH6, MLH1, and PMS2 markers. We defined dMMR as a lacking expression of one or more MMR proteins, while tumor with intact MMR proteins was categorized as pMMR.

### 2.3. Four Machine Learning Classifiers and a Conventional Logistic Regression Model

In this study, we built four machine learning models (extreme gradient boosting (XGBoost), support vector machine (SVM), naive Bayes (NB), and random forest (RF)) and a conventional logistic regression (LR) model using the caret package for R language (version 6.0-90) to diagnose dMMR discriminatively. An analysis of colinearity was performed on the initial 23 variables to exclude significantly correlated variables. Subsequently, least absolute shrinkage and selection operator (LASSO) regression and RF were used for variable selection. LASSO regression is known to be able to remove unimportant variables via the regression coefficients penalizing the size of the parameters. Applying the LASSO regression method, feature selection and predictive signature building were done. LASSO regression shrinks the coefficient estimates toward zero, with the degree of shrinkage dependent on an additional parameter, *λ*. To determine the optimal values for *λ*, a 10-time cross-validation was used. RF is an ensemble learning method based on classification and regression trees. Each tree is trained on a bootstrap sample, and optimal variables at each split are identified from a random subset of all variables. The data were randomly divided into training and validation sets by 7 : 3. The variables screened by LASSO regression and random forest methods were integrated and incorporated into the predictive models. Tenfold cross-validation and 10 × 10 grid research were used for model hyperparameter selection.

### 2.4. Data Analysis

Continuous variables between the dMMR and pMMR groups were analyzed using the Student *t*-test or the Mann–Whitney *U* test (as appropriate). Also, categorical data were compared with the chi-square test or Fisher's exact test. Receiver operating characteristic (ROC) curve was performed to assess the diagnostic performance of predictive models of dMMR. The area under the curve (AUC) was measured in each ROC curve, and specificity and sensitivity were calculated to assess the diagnostic performance of five models. The above statistical analyses were performed using the R software version. Differences were considered statistically significant when *P* < 0.05 for both sides. The Delong test was used to compare the difference in AUCs among models and a *P* value < 0.05 was considered statistically significant.

## 3. Results

### 3.1. Patient Characteristics

**Figure 1:**
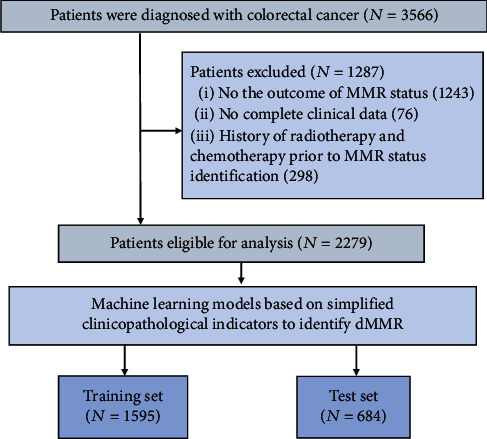
Patient screening process. The detailed process of patient selection.

We screened 3566 patients with CRC, and 2279 eligible patients were enrolled in our study. All eligible patients were recruited from Wuhan Union Medical College Hospital. In a ratio of 7 : 3, 1595 patients were allocated to the training group and 684 to the testing group. The detailed screening process is shown in Figure 1.

The demographic, clinical, and tumor-related characteristics of the patients included in the study are summarized in Table 1. Of these patients, 36.6% were younger than 53 years of age at the time of tumor development. The tumor was located in the colon in 47.5% of the patients, and 37.3% had tumors ≥ 4.6 cm in size. The tumors in 25.3% of the patients were not adenocarcinomas, 13.3% had poorly differentiated tumors, and 68.0% had tumors without PNI. Of the 2279 patients with CRC, 177 had dMMR (7.77%), and no significant difference was noticed in the incidence between men and women. Notably, younger patients with CRC were more likely to have concomitant dMMR, the diagnosis rate of which decreased with age from 10.68% before 53 years of age to 6.09% after.

Table 1 shows that the tumors were more likely to be associated with dMMR if the patients were <53 years old at presentation and the tumor was ≥4.6 cm in diameter, located in the colon, nonadenocarcinoma, poorly differentiated, TNM-stage II, and without nerve vascular invasion. If the age at presentation was <53 years and the tumor lacked neurovascular invasion, the odds increased nearly threefold; if the tumor was ≥4.6 cm in diameter, the odds increased again by a factor of 5. The ratios for the remaining features almost always ranged from 2.5–1. Table 1 also shows that the most sensitive features of dMMR were the occurrence of the tumors in the colon and the lack of nerve invasion; nearly 90% of dMMR tumors occurring in the colon lacked nerve invasion. The internal categorical distribution of each variable is shown in Supplementary Figure 1.

### 3.2. Construction of Predictive Models

**Figure 2:**
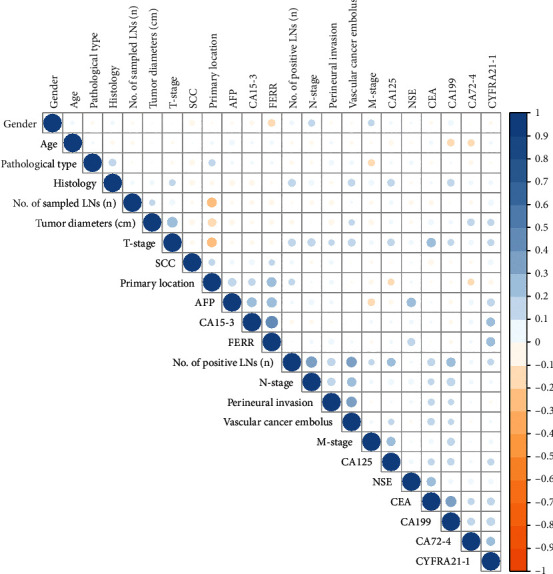
Colinearity analysis. Variables exhibiting colinearity were excluded from variate analysis. The darker blue color indicates higher colinearity.

**Figure 3:**
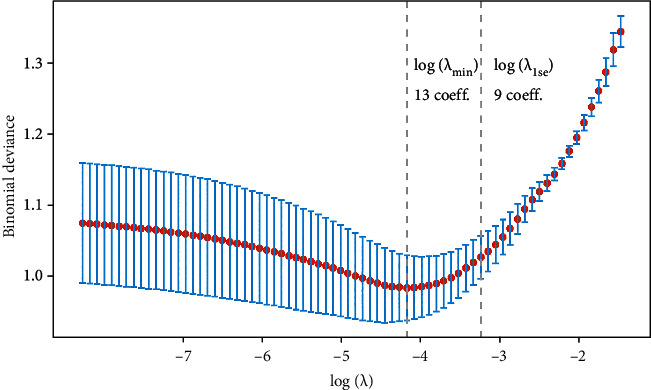
LASSO regression feature filtering. LASSO (least absolute shrinkage and selection operator) regression based on five times tenfold cross-validation was used for feature selection.

**Figure 4:**
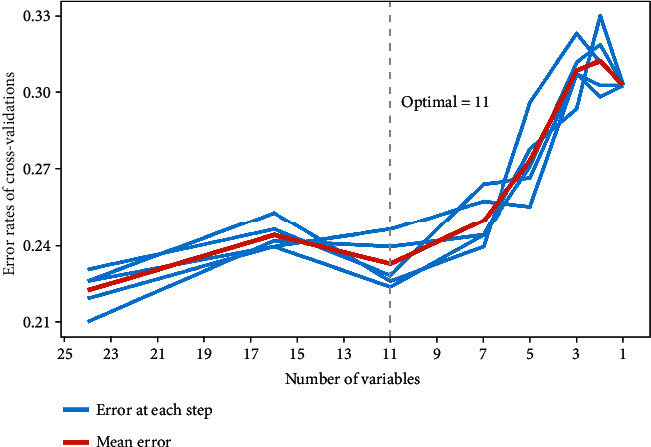
Random forest feature filtering. Random forest based on five times tenfold cross-validation was used to perform feature selection.

Twenty-three variables were initially included based on the simple clinicopathological data of the patients. The collinearity between variables was excluded before modelling. The results of the variable correlation analysis (Figure 2) showed no collinearity among the independent variables. To make the model more practical and simpler, we further selected the initial 23 variables using least absolute shrinkage and selection operator (LASSO) regression and random forest (RF) as shown in Figures 3 and 4.  Figure 3 and Supplementary Figure 2 show the same results. The *λ* value of binomial deviation under one standard error was used for the final LASSO regression by performing five times tenfold cross-validation. The LASSO regression and RF selected 9 and 11 variables, respectively. The process associated with the variable selection is shown in Supplementary Figures 3. We also combined the variables screened using both methods. The final 12 variables included in the predictive models were age, tumor location, tumor diameter, pathology type, degree of differentiation, number of lymph nodes sampled, N-stage, peripheral nerve invasion, NSE, number of positive lymph nodes, CA72.4, and TNM-stage. In total, 12 clinicopathological characteristics were used as the best subset of risk factors and as the final parameters for the model input (Table 2).

### 3.3. Performance of Models

**Figure 5:**
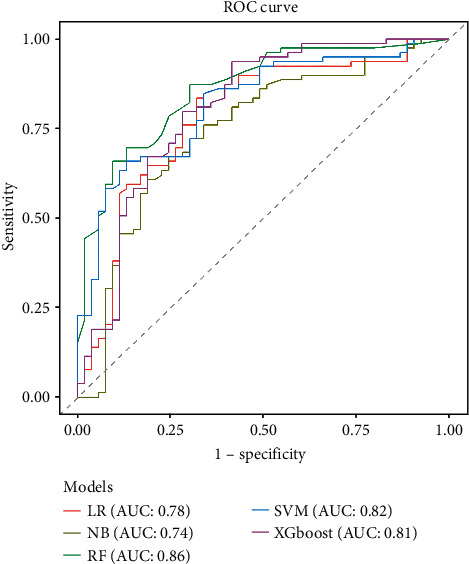
Receiver operating characteristic (ROC) curves of predictive models. Diagnostic abilities of predictive models for the differential diagnosis of dMMR and pMMR in the test set. ROC curves of predictive model created by LR, NB, RF, SVM, and XGBoost.

The 2279 patients with CRC were randomly divided into training and test sets in a 7 : 3 ratio. The receiver operating characteristic (ROC) curve was used to evaluate the performance of the four machine learning models and LR model. As shown in Figure 5, the area under curve (AUC) values of the test set were as follows: XGBoost: 0.8055; SVM: 0.8174; NB: 0.7424; RF: 0.8584; and LR: 0.7835 (Delong test, *P* value < 0.05). Therefore, we concluded that the RF model has an excellent predictive ability to identify CRC with dMMR with a sensitivity of 0.8679 and a specificity of 0.6962. Moreover, machine learning models showed a better predictive ability than that of the conventional LR method. The accuracy, sensitivity, specificity, positive predictive value (PPV), and negative predictive value (NPV) of the predictive models on the test set are listed in Table 3.

### 3.4. Variable Importance Analysis

**Figure 6:**
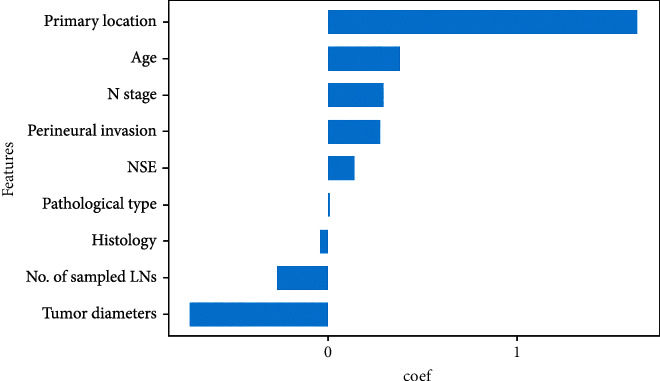
Significance analysis by LASSO regression. For the LASSO (least absolute shrinkage and selection operator) regression, we give the normalized regression coefficients for each feature.

**Figure 7:**
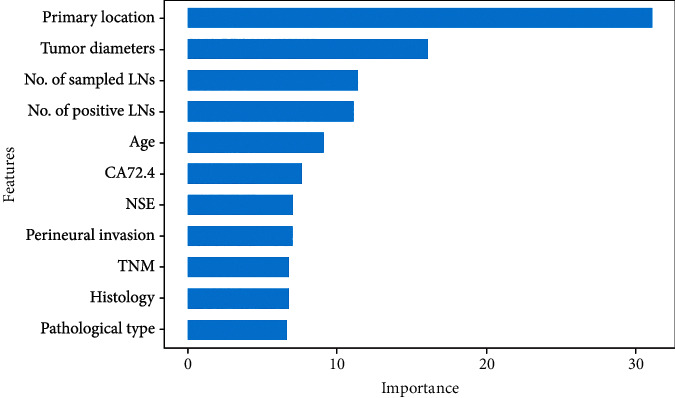
Significance analysis by Random forest. We used the machine learning technique and random forest, to determine feature importance.

**Figure 8:**
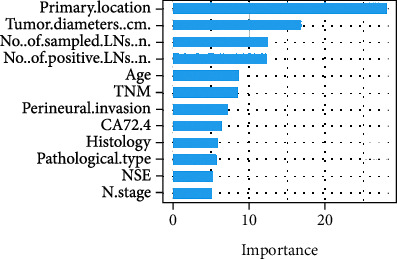
Variable importance analysis. The merged variables were performed for feature importance analysis by random forest.

We performed feature importance analysis for the variables selected using LASSO regression and random forest, respectively, and the results are displayed in Figures 6 and 7. The final 12 variables were incorporated into the predictive models for training and testing. To investigate the potential impact of each clinical feature on the recognition ability of the predictive model, we ranked the clinical variables that showed the best results in the RF model in order of their contribution to the output results from highest to lowest, as shown in Figure 8. We found that the location and diameter of the tumor were placed in the top two rankings, which was same as in the results shown in Figures 6 and 7. This means that when a patient has a tumor in the colon that is ≥4.6 cm in diameter, the tumor is more likely to be associated with dMMR or MSI status.

## 4. Discussion

CRC remains a major healthcare burden with a high mortality rate worldwide [17]. MMR plays a key role in CRC progression and prognosis. The latest guidelines recommend chemotherapy for patients with stage II CRC with proficient MMR (pMMR) even without high-risk factors [18]. The current rapid advancement of medical science enables genetic testing techniques to be applied to MMR status to optimize personalized treatment and management of CRC in patients. However, the diagnosis rate of MMR status is still not high [19–21]. A number of artificial intelligence diagnostic models are currently available to predict MMR/MSI status with good results, but the predictions are based on identifying pathological sections. We compiled our own database to build multiple machine learning models to predict MMR/MSI using simple clinicopathological indicators, covariance analysis results, LASSO regression feature screening, and RF feature screening to select appropriate variables. We used AUC values to assess the discrimination ability of the machine learning models. The results showed that machine learning models built on simple clinicopathological indicators could accurately predict the MMR/MSI status of patients.

In previous studies, [5] we analyzed clinicopathological data of 3274 participants from two institutions and assessed their predictive value in patients of all ages with CRC. We found that a columnar line graph created using simple clinicopathological indicators was able to accurately predict the status of MMR/MSI in patients with an AUC value of 0.754 (95% CI: 0.715–0.793) in the validation group. Notably, the addition of serum tumor markers CEA and CA72-4 to the model increased the AUC value in the validation group to 0.796 ((0.758–0.835), *P* < 0.001). Cross-validation, calibration curves, and decision curves validated the predictive accuracy of the column-line graphs. In this study, we further focused on whether the machine learning approach was more effective than that of the conventional predictive model by constructing machine learning models based on simple clinicopathological indicators. Satisfactorily, the five predictive models that we built achieved better identification results overall: XGBoost: 0.8055; SVM: 0.8174; NB: 0.7424; RF: 0.8584; and LR: 0.7835 in the test group.

Few studies have built machine learning models based on simple clinicopathological indicators to predict dMMR or MSI status in patients with CRC. Notably, several studies have been reported to use deep learning methods based on pathologically stained images to identify the MMR/MSI status in patients with CRC. Echle et al. [2] built a deep learning classifier to detect MMR/MSI status in tumor samples based on conventional histology slides that were obtained from 8836 patients with CRC from Germany, the UK, the USA, and the Netherlands. They conducted separate experiments to determine the appropriate sample size for the training set and indicated that color-normalized images would increase the recognition rate of MMR/MSI states. Meanwhile, they used an external validation cohort to verify their findings. More importantly, they also explored the use of endoscopic biopsy samples to identify the MMR/MSI status. The major part of this experimental study comprised of the 6406 patients in the training group and 771 patients in the external validation group. The validation group had an AUC value of 0.95 (range, 0.92–0.96), which increased to 0.96 (range, 0.93–0.98) after picture processing. Jiang et al. [22] developed a deep learning model to predict the DNA MMR status in CRC based on HE-stained whole slide images (WSI). Though the study by Echle et al. had a much larger sample size and was a multicenter study, this study proposes a dual-threshold triage strategy that can be used to exclude patients with dMMR, which increases the surgical and biopsy specimen-sensitivity to >90% and specificity to >95% for the triage of patients with MMR. In this study, the AUC values were 0.8888 ± 0.0357 for TCGA validation cohort, 0.8806 ± 0.0232 for the pathology AI platform validation cohort, 0.8457 ± 0.0233 for the SYSUCC surgery cohort, and 0.7679 ± 0.0342 for the SYSUCC biopsy cohort. Despite the relatively low AUC value, it remains a good result, and the clinical significance of the dual-threshold triage strategy remains very high. Schrammen et al. [23] developed a slide-level assessment model (SLAM) based on HE-stained slides. This was also a multicenter study with a sample size of approximately 3300 participants and an AUC value of 0.9000 for the external validation group. Notably, this study proposes a three-variable visualization approach to improve the interpretability of the model. In addition, the model can also identify the BRAF mutation status. Several similar studies have been conducted [24–26].

The development of scoring systems has also been effective in predicting the dMMR or MSI status in patients with CRC. Jenkins et al. [27] used multiple linear logistic regression to develop the MsPath scoring system to identify the patients with dMMR or MSI-H status and give appropriate recommendations for performing further testing. The model included indicators of diagnosis before the age of 50, tumor located on the right, mucinous carcinoma, low differentiation, Crohn's-like lymphocytic response, and tumor-infiltrating lymphocytes (TIL), with the highest correlation coefficient for the TIL indicator. The development and validation of separate models for different ethnic populations have increased the applicability of these models. Furthermore, the scoring system provided a clear recommendation for the patient regarding the need for further testing. Additionally, the scoring is simple to perform and requires only some simple clinicopathological data for the initial assessment. However, this scoring system is not effective for patients with CRC above 60 years of age. Nevertheless, compared with that in our study, this scoring system outperformed the RF model in terms of predictive performance (AUC : 0.89 > AUC : 0.8584), and it incorporated only half as many features as we did. The inclusion of different geographical populations has added to the usefulness of the model. More importantly, those models were easier to interpret than ours were. A similar scoring model was developed by Greenson et al. [28] using LR. The characteristics were the same, except for the presence or absence of organ necrosis. This scoring system included patients with CRC above 60 years of age and had an AUC of 0.85. Other similar models include the PREDICT model [29] and RERtest6 and RERtest 8 models [30].

Our previously established scoring model incorporated nine clinicopathological features: age, location, tumor diameter, degree of differentiation, number of sample lymph nodes, PNI, number of positive lymph nodes, CA72-4, and CEA. The machine learning model built in this study excluded the CEA feature and included the three features NSE, N staging, and TNM staging, which were the results of a series of feature screenings. Compared with similar studies, we included a relatively large number of features, but the predictive performance of the model did not correlate with the number of indicators included. Of these, tumor location and size contributed most to the model. The comparison revealed that four characteristics—age, tumor location, Crohn's-like reaction, and TIL—were frequently included in the scoring model and had high coefficients. The difference is that although in the current study the tumor location is divided into rectum and colon, it was not additionally classified into proximal, distal, or left and right; nor were the two features Crohn's-like reaction and TIL included.

We analyzed the strengths and weaknesses of each model. Support vector machines have the advantage of being able to perform linear and nonlinear classification and regression but struggle to deal effectively with complex and large data. RF and gradient boosting (e.g., XGBoost) have the advantage of being able to understand the importance of each feature for prediction, explain how decisions are made, and are easier to train and tune. The disadvantage is that these models are unsuitable for regression [31]. The classification efficiency of plain leaf bass is more stable and suitable for handling small-scale data, but it assumes that the features are independent of each other. Hence, this model is not applicable for the analysis of our data. For example, adenocarcinoma is associated with age, and older patients are more likely to develop colorectal adenocarcinoma [32]. This may be the reason for the lowest predictive effect among the five models.

Current research on the prediction of MMR status in colorectal cancer has two main approaches. One is to build a deep learning model to predict the MMR status by identifying the pathologically stained sections. The second is to build scoring models, filter the final incorporated features of the models through univariate and multivariate analyses, and then predict the MMR status. The novelty of this study is the combination of artificial intelligence methods and simple clinicopathological indicators to predict the status of MMR in patients with CRC.

## 5. Conclusions

In this study, we built four sets of machine learning models and a conventional logistic regression model to predict the lack of DNA MMR in patients with CRC based on simple clinicopathological indicators. Our results show that machine learning models can be incorporated with accurate and consistent predictive behavior. In fact, machine learning models show better performance at identifying dMMR than the conventional logistic regression methods. To the best of our knowledge, this study is the first to propose a machine learning approach to analyze and model the MMR status of patients based on simple clinicopathology and tumor markers. In addition, our single-center sample was sufficiently large to draw conclusions with some reference values. In future studies, we aim to incorporate TIL and Crohn's-like response features into the prediction model, refine some of the features such as the location of the tumor, and add an external validation group to improve the predictive power of the model.

## 6. Limitations

Our study has some limitations. First, the population in our cohort comprised of persons from one region of China (Wuhan), which may limit the generalizability of the predictive models and require further validation in patients from different geographic regions. Second, this was a nonrandomized retrospective analysis. Therefore, potentially biased comparisons such as in the inclusion of patients or sample selection bias could have occurred. Third, the included indicators need to be refined; for example, the location of the tumor needs to be subdivided into left or right and proximal or distal colon. Crohn's-like reaction and tumor lymphocyte infiltration also need to be included in the model. Finally, we only performed internal validation in this study, and further external validation groups are needed to verify the predictive effect.

## Figures and Tables

**Table 1:** Clinical characteristics of the patients with colorectal cancer.

Level	*n*	Overall	dMMR	PMMR	*P* value
		2279	177	2102	
Gender (%)	Male	1369 (60.1)	107 (60.5)	1262 (60.0)	0.978
	Female	910 (39.9)	70 (39.5)	840 (40.0)	
Age (%)	<53	833 (36.6)	89 (50.3)	744 (35.4)	<0.001
	≥53	1446 (63.4)	88 (49.7)	1358 (64.6)	
Primary location (%)	Colon	1082 (47.5)	159 (89.8)	923 (43.9)	<0.001
	Rectum	1197 (52.5)	18 (10.2)	1179 (56.1)	
Tumor diameters (cm (%))	<4.6	1420 (62.3)	52 (29.4)	1368 (65.1)	<0.001
	≥4.6	859 (37.7)	125 (70.6)	734 (34.9)	
Pathological type (%)	Nonadenocarcinoma	576 (25.3)	61 (34.5)	515 (24.5)	0.01
	Adenocarcinoma	1703 (74.7)	116 (65.5)	1587 (75.5)	
Histology (%)	Moderate	1978 (86.7)	137 (77.4)	1839 (87.5)	<0.001
	Poor	303 (13.3)	40 (22.6)	263 (12.5)	
No. of sampled LNs (m (%))	<23	1735 (76.1)	85 (48.0)	1650 (78.5)	<0.001
	≥23	544 (23.9)	92 (52.0)	452 (21.5)	
No. of positive LNs (*n*) (mean (SD))		2.02 (3.85)	0.89 (2.90)	2.12 (3.90)	<0.001
T-stage (%)	I/II	405 (17.8)	20 (11.3)	385 (18.3)	0.025
	III/IV	1874 (82.2)	157 (88.7)	1717 (81.7)	
N-stage (%)	N0	1249 (54.8)	134 (75.7)	1115 (53.0)	<0.001
	N2	430 (18.9)	11 (6.2)	419 (19.9)	
	N1	600 (26.3)	32 (18.1)	568 (27.0)	
M-stage (%)	0.00	2237 (98.2)	174 (98.3)	2063 (98.1)	1.00
	1.00	42 (1.8)	3 (1.7)	39 (1.9)	
TNM (%)	1.00	319 (14.0)	18 (10.2)	301 (14.3)	<0.001
	2.00	913 (40.1)	115 (65.0)	798 (38.0)	
	3.00	1005 (44.1)	41 (23.2)	964 (45.9)	
	4.00	42 (1.8)	3 (1.7)	39 (1.9)	
Perineural invasion (%)	No	1549 (68.0)	160 (90.4)	1389 (66.1)	<0.001
	Yes	730 (32.0)	17 (9.6)	713 (33.9)	
Vascular cancer embolus (%)	No	1734 (76.1)	146 (82.5)	1588 (75.5)	0.047
CA72-4(%)	Normal	1891 (83.0)	120 (67.8)	1771 (84.3)	<0.001
	High	388 (17.0)	57 (32.2)	331 (15.7)	
CA199 (%)	Normal	1855 (81.4)	144 (81.4)	1711 (81.4)	1.000
	High	424 (18.6)	33 (18.6)	391 (18.6)	
AFP	Low	298 (13.1)	34 (19.2)	264 (12.6)	0.036
	Normal	1957 (85.9)	142 (80.2)	1815 (86.3)	
	High	24 (1.1)	1 (0.6)	23 (1.1)	
SCC (%)	Normal	2174 (95.4)	168 (94.9)	2006 (95.4)	0.897
	High	105 (4.6)	9 (5.1)	96 (4.6)	
NSE (%)	Normal	1513 (66.4)	122 (68.9)	1391 (66.2)	0.508
	High	766 (33.6)	55 (31.1)	711 (33.8)	
CA125 (%)	Normal	2060 (90.4)	157 (88.7)	1903 (90.5)	0.508
	High	219 (9.6)	20 (11.3)	199 (9.5)	
CA15-3 (%)	Normal	872 (38.3)	83 (46.9)	789 (37.5)	0.017
	High	1407 (61.7)	94 (53.1)	1313 (62.5)	
FERR (%)	Low	1134 (49.8)	123 (69.5)	1011 (48.1)	<0.001
	Normal	1018 (44.7)	49 (27.7)	969 (46.1)	
	High	127 (5.6)	5 (2.8)	122 (5.8)	
CYFRA21-1 (%)	Normal	1706 (74.9)	132 (74.6)	1574 (74.9)	1.000
	High	573 (25.1)	45 (25.4)	528 (25.1)	

**Table 2:** Risk factors for deficient MMR in colorectal cancer. Five predictive models based on simplified clinicopathological features and serum tumor biomarkers.

Characteristic	OR^1^	95% CI^1^	*P* value
*Age*			<0.001
<53	—	—	
≥53	2.93	1.54, 5.72	
*Primary location*			<0.001
Colon	—	—	
Rectum	10.5	4.95, 23.9	
*Tumor diameters (cm)*			<0.001
<4.6	—	—	
≥4.6	0.24	0.12, 0.46	
*Pathological type*			0.23
Nonadenocarcinoma	—	—	
Adenocarcinoma	1.53	0.76, 3.10	
*Histology*			0.21
Well/moderate	—	—	
Poor	0.59	0.25, 1.35	
*No. of sampled LNs(n*)			0.009
<23	—	—	
≥23	0.42	0.21, 0.80	
*N-stage*			0.76
N0	—	—	
N1	2.67	0.02, 515	
N2	5.58	0.02, 1683	
*Perineural invasion*			0.028
No	—	—	
Yes	2.87	1.12, 7.85	
*NSE*			0.018
Normal	—	—	
High	2.20	1.15, 4.33	
*No. of positive LNs(n*)	1.14	0.87, 1.73	0.47
*CA72.4*			0.23
Normal	—	—	
High	0.65	0.31, 1.32	
*TNM*			0.71
1	—	—	
2	1.86	0.65,5.34	
3	1.30	0.01, 195	
4	1.24	0.02, 90.2	

^1^OR: odds ratio, CI: confidence interval.

**Table 3:** Performance of different predictive models to identify dMMR. Accuracy, sensitivity, specificity, positive predictive value, and negative predictive value of five predictive models in the test set.

Model	Sensitivity	Specificity	Pos Pred value	Neg Pred value	Accuracy	AUC-ROC
LR	0.7170	0.7595	0.6667	0.8000	0.7424	0.7835
RF	0.8679	0.6962	0.6571	0.8871	0.7652	0.8584
NB	0.7547	0.6329	0.5797	0.7937	0.6818	0.7424
SVM	0.7736	0.6709	0.6119	0.8154	0.7121	0.8174
XGBoost	0.7170	0.7468	0.6552	0.7973	0.7348	0.8055

## Data Availability

The datasets used and/or analyzed during the current study are available from the corresponding authors on reasonable request.

## Supplementary Materials

Supplementary 1 Analytical diagram of the percentage of the situation within each variable. We performed a statistical analysis of the individual signs of the included patients, which is presented in the form of a bar chart that clearly shows the proportion of the number of patients for each variable.

Supplementary 2 Display of variable coefficients in logistic regression model. For the logistic regression model, the coefficients assigned to each feature were recorded.

Supplementary 3 LASSO regression feature filtering. LASSO (least absolute shrinkage and selection operator) regression based on five times tenfold cross-validation was used for feature selection.
