# Supplementary figures and images for: Development and Interpretation of a Clinicopathological-Based Model for the Identification of Microsatellite Instability in Colorectal Cancer
Source: Dis Markers. 2023 Feb 18;2023:5178750. doi: 10.1155/2023/5178750 (PMC9969972; doi:10.1155/2023/5178750)

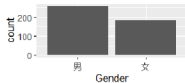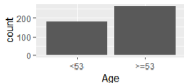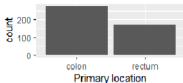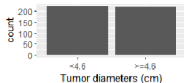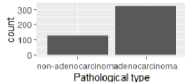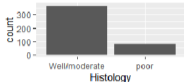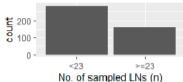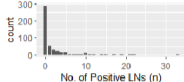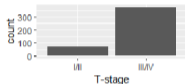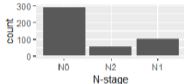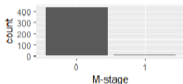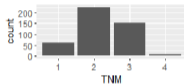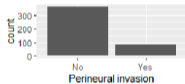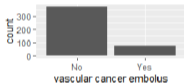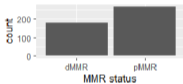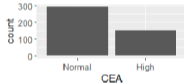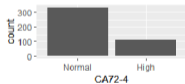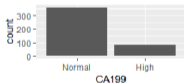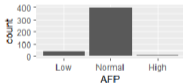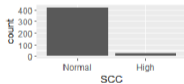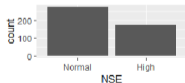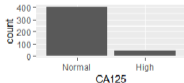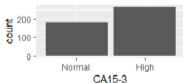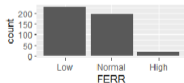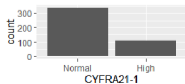

Supplement: Supplementary 1 — Analytical diagram of the percentage of the situation within each variable. We performed a statistical analysis of the individual signs of the included patients, which is presented in the form of a bar chart that clearly shows the proportion of the number of patients for each variable. [file 5178750.f1.pdf]

features

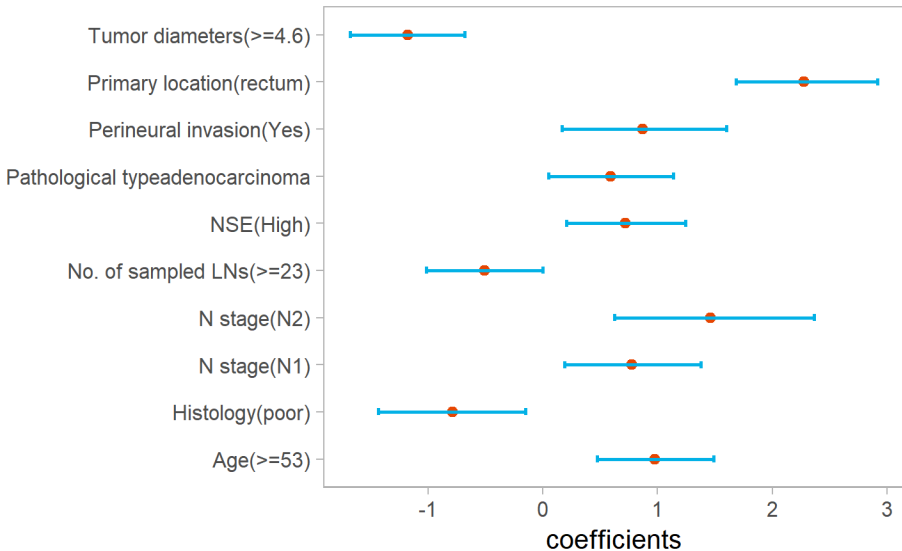

Supplement: Supplementary 2 — Display of variable coefficients in logistic regression model. For the logistic regression model, the coefficients assigned to each feature were recorded. [file 5178750.f2.pdf]

Binomial Deviance

24 23 23 21 20 19 20 17 16 13 10 9 6 4 2 1

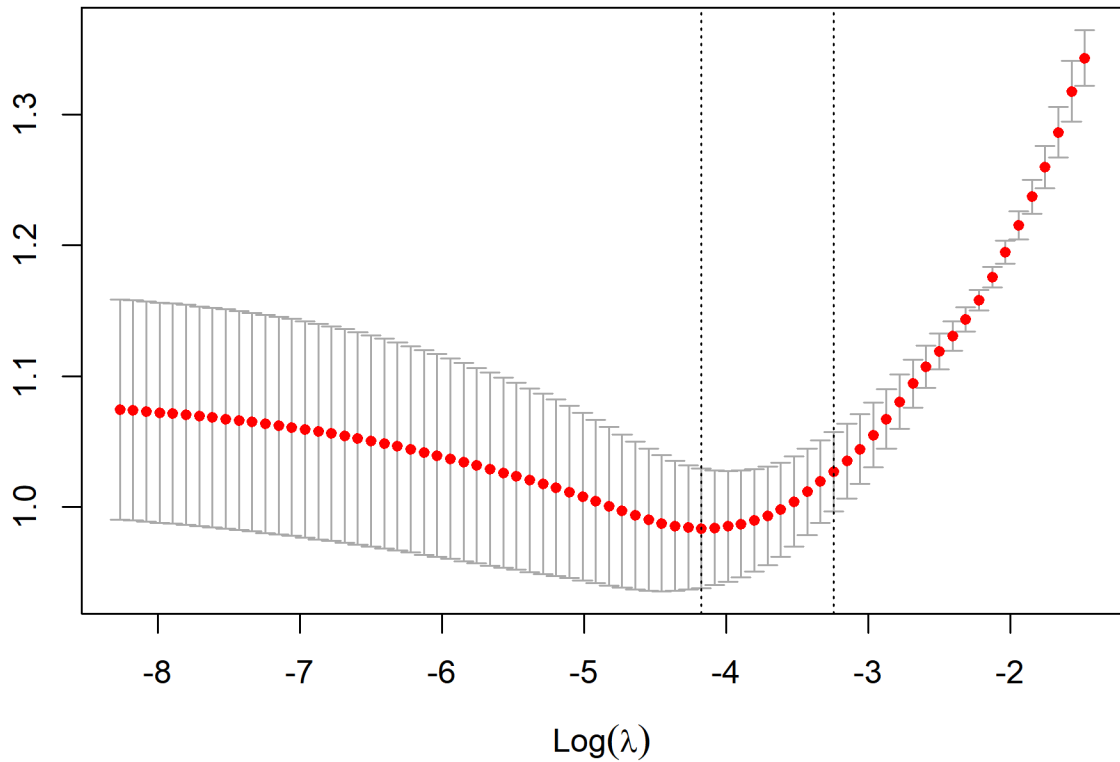

Supplement: Supplementary 3 — LASSO regression feature filtering. LASSO (least absolute shrinkage and selection operator) regression based on five times tenfold cross-validation was used for feature selection. [file 5178750.f3.pdf]
